# Localized Delivery of a Small Molecule Hedgehog Agonist via Poly(ε‐caprolactone) Scaffolds Enhances Tendon‐to‐Bone Integration

**DOI:** 10.1002/jor.70183

**Published:** 2026-03-30

**Authors:** Jonathan Marcelin, Rashad Madi, Timur B. Kamalitdinov, Xi Jiang, Dong Hwa Kim, Robert L. Mauck, Andrew F. Kuntz, Nathaniel A. Dyment

**Affiliations:** ^1^ Department of Orthopaedic Surgery University of Pennsylvania Philadelphia Pennsylvania USA; ^2^ Department of Bioengineering University of Pennsylvania Philadelphia Pennsylvania USA; ^3^ Translational Musculoskeletal Research Center Corporal Michael J. Crescenz Veterans Administration Medical Center Philadelphia Pennsylvania USA

**Keywords:** bone tunnel integration, hedgehog signaling, mineralized fibrocartilage, scaffold drug delivery

## Abstract

Tendon and ligament injuries often require surgery, with successful recovery, whether direct repair of a torn tendon to bone or reconstruction of ligaments, requiring effective tendon‐to‐bone integration. Unfortunately, re‐tear rates can be high, demonstrating the need for improved treatment strategies. The Hedgehog (Hh) pathway is critical to fibrocartilage formation at tendon and ligament insertion sites during development, and recent studies indicate that it promotes tendon‐to‐bone integration post‐surgery. However, systemic delivery of Hh signaling agonists can result in off‐target effects. In this study, we developed a scaffold system for local delivery of a Hh signaling agonist (SAG) to promote tendon‐to‐bone integration. We fabricated aligned electrospun scaffolds loaded with four concentrations of SAG and quantified the fiber alignment and diameter, which were not different between the different concentrations. We then measured the in vitro release profile and found a dose‐dependent release of SAG from the scaffolds, as measured by expression of the downstream Hh target gene, Gli1, in bone marrow stromal cell cultures that received conditioned media. Finally, we combined the scaffolds with tendon grafts and inserted them into bone tunnels created in the proximal tibiae of mice. SAG released from the scaffolds did not affect cell infiltration in the graft or scaffold, but increased expression of Gli1 by cells in the bone tunnels while also increasing mineralized fibrocartilage formation in a dose‐dependent manner. These findings indicate that scaffold‐mediated local delivery of SAG can effectively enhance tendon‐to‐bone integration, offering a promising strategy for treating tendon and ligament injuries.

## 1 Introduction

Tendon and ligament injuries represent a significant socioeconomic burden, affecting approximately one‐third of the adult population in the United States [1]. These injuries frequently occur near the insertion site into bone, also known as the enthesis, and often necessitate surgical intervention, such as rotator cuff repair in the shoulder or anterior cruciate ligament reconstruction (ACLR) in the knee. As successful repair outcomes to these surgeries require tendon‐to‐bone integration, it is essential to understand how to recreate a zonal tendon‐to‐bone insertion site following surgical repair. Unfortunately, re‐tear rates for these procedures can be considerably high, especially for large tear sizes in the rotator cuff and in young athletes following ACL reconstruction [2]. This highlights the need to develop novel therapeutics to enhance tendon‐to‐bone integration and improve repair outcomes.

One strategy to improve tendon‐to‐bone repair is to identify pivotal signaling pathways in the formation of this intricate tissue. Several pivotal studies demonstrated a critical role for hedgehog (Hh) signaling in the development of the zonal enthesis [3, 4, 5, 6]. In one study, it was shown that the Hh signaling transcription factor Gli1 (Glioma‐Associated Oncogene Homolog 1), often indicative of cells with active Hh signaling, was expressed by cells at the interface between the tendon/ligament midsubstance and the underlying nascent cartilage at embryonic and early postnatal stages [6]. These Gli1‐expressing cells then gave rise to fibrochondrocytes in the enthesis at later stages. Gain‐ and loss‐of‐function studies also demonstrated that the Hh signaling pathway promoted the production and maturation of the fibrocartilage zones within the enthesis [3, 4, 5, 6]. These developmental studies indicated that the Hh pathway could be a potent target for improving zonal fibrocartilaginous entheses during tendon‐to‐bone repair in the adult.

Given these findings during growth and development, several groups have since investigated a potential role for modulation of the Hh pathway to promote tendon‐to‐bone repair. Hh signaling is active during the healing process, as demonstrated following injury of the rotator cuff [7, 8, 9] and during the tendon‐to‐bone integration process within the bone tunnels following anterior cruciate ligament reconstruction (ACLR) [10, 11]. Our group recently stimulated the pathway genetically (with an over‐expressing genetic mouse model) and pharmacologically (via systemic injections of a Hh agonist) and found that pathway activation promoted tendon‐to‐bone attachment formation in the ACLR model [12]. Similar positive effects were also found in the rotator cuff [7]. These findings indicate that modulation of Hh signaling is a promising target for improving tendon‐to‐bone repair. However, it is well known that Hh plays a key role in the maintenance of many tissues and organs [13], and genetic alterations of the pathway in certain cell types have led to adverse effects such as osteoarthritis [14, 15], hair loss [16, 17], ectopic mineralization [18], and certain cancers [19]. In fact, we previously reported hair loss in a subset of mice receiving system agonist injections over a number of weeks and ectopic mineralization in a genetic activation model [12]. Therefore, there is a clear need to develop a method to provide localized therapy to avoid off‐target effects.

To that end, in the current study, we fabricated electrospun poly(*ε*‐caprolactone) (PCL) scaffolds loaded with several concentrations of the Hh signaling agonist (smoothened agonist, SAG). These scaffolds not only provide a template for organized neotissue formation, but can also be leveraged to release small molecules directly or indirectly [20, 21, 22, 23]. We first measured alignment and diameter of the electrospun fibers to determine if SAG incorporation had an effect on the scaffold structural properties. Next, we measured the in vitro release and bioactivity of SAG released from the scaffolds. Finally, we combined the scaffolds with tendon grafts and inserted them into bone tunnels created in the proximal tibiae of mice to determine if release of SAG affected the tendon‐to‐bone integration process in the tunnel. Our hypothesis was that localized delivery of SAG from the scaffold would improve the tendon‐to‐bone integration process by promoting the production of mineralized fibrocartilage attachments.

## 2 Methods

### 2.1 Experimental Design

**Figure 1:**
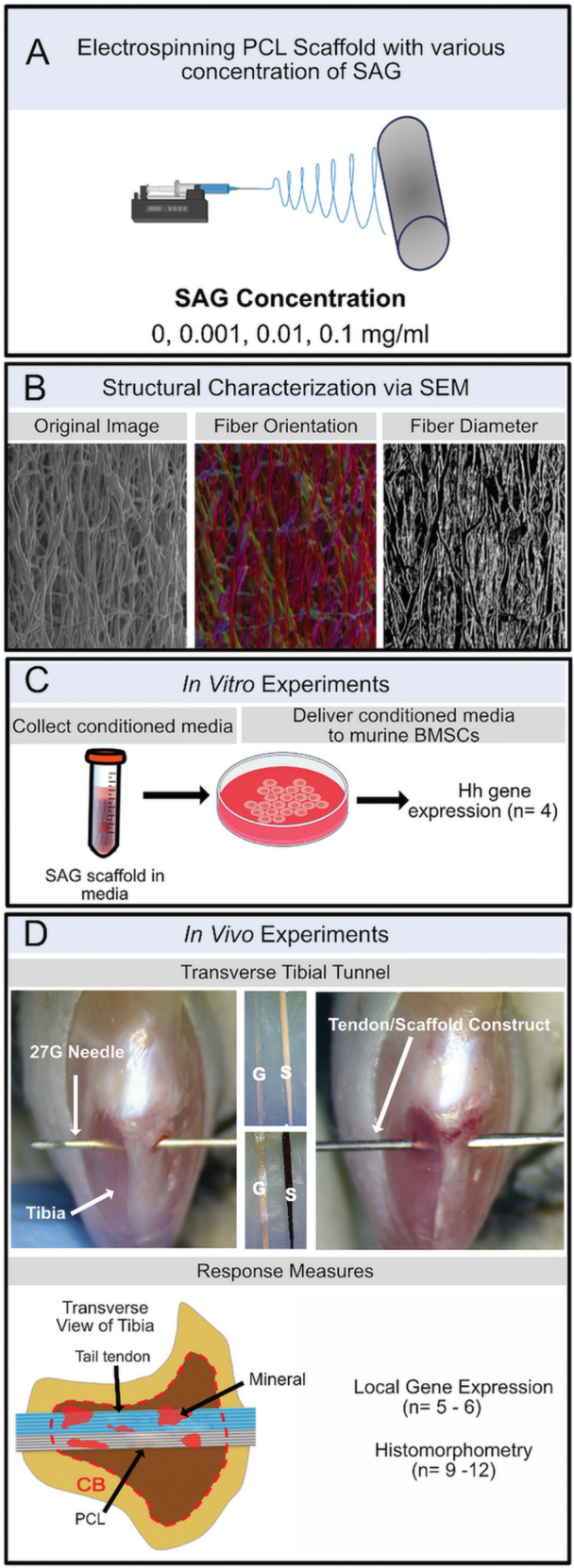
Experimental design. (A) Electrospinning of blank (0 mg/mL) and SAG‐loaded PCL scaffolds followed by (B) analysis of scaffold fiber alignment and diameter. (C) In vitro release study: SAG‐loaded scaffolds were placed in media for 26 days, and conditioned media was then given to bMSCs in culture. Gli1 gene expression was quantified via qPCR. (D) In vivo assessment: During the procedure, electrospun PCL scaffolds (dyed black for visualization purposes) were combined with fascicles from a tail tendon autograft to form a tendon‐scaffold construct that was passed through the tibial tunnel, enabling local SAG release from the nanofibers. At 14 days post‐surgery, microdissected tissues were analyzed for Gli1 expression, and mineral deposition within the tendon graft and scaffold in the bone tunnels was assessed. G: Tendon graft; S: Scaffold.

Aligned electrospun PCL scaffolds loaded with 0, 0.001, 0.01, 0.1 mg/mL of SAG were fabricated (Figure 1). The structure of the scaffolds was assessed via SEM. Next, an in vitro release study was conducted where conditioned media from the scaffolds were delivered to bone marrow stromal cells (bMSCs) in culture (*n* = 4/group) to determine if the released SAG remained bioactive and if it activated Hh signaling in the cells in a dose‐dependent manner. Finally, bilateral transverse tibial tunnels (TTTs) were created in the proximal metaphyses of 24 mice (mean ± SD age, 18.5 ± 3.7 weeks old; mean ± SD bodyweight, 34.2 ± 7.9 g), where each limb received a scaffold from one of the four SAG concentrations. The four groups were equally distributed among the limbs to make the intra‐animal comparisons between the groups equally weighted and to minimize surgical variability that may occur between right and left limbs. To measure the expression of Hh‐ and enthesis‐related genes, the mice were sacrificed at 14 days post‐surgery for quantitative real‐time PCR (qPCR) analysis from microdissected histological sections (*n* = 5–6/group) as well as histological analysis (*n* = 9–12/group). Both male and female mice of a mixed CD1;C57BL/6 background were included (14 males and 10 females). All animals and procedures were approved by the IACUC (Protocol #806077) at the University of Pennsylvania.

### 2.2 Scaffold Fabrication

Poly(*ε*‐caprolactone) (PCL) nanofibrous scaffolds (PCL, mol. wt. 80 kDa, Shenzhen Bright China Industrial Co. Ltd., China) were fabricated via electrospinning as described previously [20, 24, 25]. Briefly, to create approximately 300 μm thick scaffolds, SAG (Selleck Chemicals Catalog No. S7779, Houston, TX, USA) was dissolved in the PCL solution (35% wt/vol in 1:1 tetrahydrofuran and N,N‐dimethylformamide) at 0, 0.001, 0.01, or 0.1 mg/mL (0, 1.9, 19.0, 189.9 μM, respectively). These concentrations were estimated based on preliminary studies where SAG was introduced to culture media to stimulate Hh signaling in bMSCs (Figure S1). The solution (2 mL) was then loaded into a 10 mL syringe and extruded (2.5 mL/hour) through an 18 G stainless‐steel needle (charged to +15 kV) 15 cm away from the rotating mandrel. The temperature and humidity controlled electrospinning room was set to 25⁰C and 35% humidity. Fibers were collected on the rotating collecting mandrel (Figure 1) to form aligned scaffolds (surface velocity of ~8 m/s). Scaffolds were sterilized with 20 min exposure to UV light on each side of the scaffold, followed by a 15 min incubation in 75% EtOH. The scaffolds were then washed 3X in sterile PBS prior to being used in the studies described below.

### 2.3 Scaffold Structural Analysis

To determine if the incorporation of SAG in the spinning solution had an effect on the overall structure of the scaffold, morphological analysis was carried out using a scanning electron microscope (SEM, FEI Quanta 600 ESEM). Lower magnification images (1000×) were taken at multiple locations to qualitatively assess the homogeneity of the scaffold. Fiber diameter and fiber alignment were calculated from 4 images (2574×) from these locations using the General Image Fiber Tool (GIFT) [26] and OrientationJ plugin in FIJI (Figure 1). The fiber diameter measurements were randomly downsampled to 500 points per group to facilitate plotting.

### 2.4 In Vitro Release Study

Both the blank and SAG‐loaded scaffolds (44.84 cm^2^, 52.8% of total area of spun scaffold) were placed in culture media (DMEM, 5% FBS, 1% pen/strep) in 50 mL tubes covered in aluminum foil on a shaker at 37⁰C for 26 days. Conditioned media (CM) was collected every other day by removing one half of the volume, which was replaced with fresh media, on days 2, 4…26, and then frozen at −80⁰C. Bone marrow stromal cell (bMSC) cultures were prepared by flushing the bone marrow from the femora and tibiae of four 6‐week‐old CD1 mice. These cells were expanded and then passaged into 24‐well plates. CM collected from each scaffold on days 2, 8, 16, or 26 was then delivered to the bMSCs for 4 days with a media change on day 2 (Figure 1). CM samples were compared to positive control groups, which received 3 nM or 300 nM of SAG added directly to the culture media. Negative controls consisted of media without SAG supplementation. RNA was isolated from the cells and qPCR (normalized to the 18S housekeeping gene) was conducted for the downstream Hh gene, Gli1, to determine the extent to which SAG released from the scaffolds induced downstream Hh signaling.

### 2.5 In Vivo assessment of SAG release on tendon‐to‐bone integration

Following the in vitro release study, TTT surgeries were performed on 12 mice bilaterally to test the effect of SAG release on stimulating Hh activity by cells within the bone tunnel and on tendon‐to‐bone integration. This procedure was utilized as a simpler, higher‐throughput model that allows for bilateral surgeries, unlike the ACLR model that our group has used previously [10, 12]. The treatment groups were equally weighted between left and right limbs to avoid surgical bias between limbs and each group had the same number of intra‐animal comparisons with each other group (*n* = 12 limbs/group). Ethiqa XR (buprenorphine extended‐release, 1.3 mg/mL; 3.25 mg/kg subcutaneous) was administered preoperatively, providing therapeutic analgesia for up to 72 h post‐surgery. Tail tendon grafts (~1 mm diameter) were harvested from 24 mice, as previously described [12]. During the procedure, a sterilized scaffold (1 mm width) was added to the graft bundle, a transverse tunnel was hand drilled in the proximal metaphyseal region of the tibia with a 27 G needle, and the resulting tendon‐scaffold construct was then passed through the tibial tunnel (Figure 1D). The animals were observed daily for 3 days followed by weekly until sample harvest at day 14 post‐surgery.

### 2.6 qPCR of Microdissected Tissue

Following euthanasia, hindlimbs were harvested and fixed in formalin for 2 days, transferred to 30% sucrose overnight, and embedded in OCT. Tape‐stabilized (Cryofilm type 2 C, Section Lab, Japan) 8μm thick frozen mineralized transverse sections of the tibia through the bone tunnels were collected. The sections (*n* = 6 per sample) were kept frozen on a chilled slide while a 27 G needle was used to cut out the region of the section containing the bone tunnel and rest of the medullary canal. The collected tissue from multiple samples was collated and then digested in Proteinase K (Zymo, D3001‐2‐5) digestion buffer (Zymo D3050‐1‐5) for 1 h at 55°C. RNA was isolated using the Quick‐RNA MicroPrep (Zymo, R1050) spin columns, converted to cDNA (Invitrogen SuperScript IV VILO), and amplified (Preamp master mix, Standard BioTools) for Gli1 (Mm00494654_m) as previously described [27]. Following this amplification step, qPCR for Gli1 was conducted and normalized to the 18S (Mm03928990_g1) housekeeping gene.

### 2.7 Multiplexed Mineralized Cryohistology to Assess Tunnel Integration

Sections adjacent to those used for qPCR were subjected to three rounds of staining and imaging on the Zeiss Axio Scan.Z1 digital slide scanner. In the first found, the section was stained with Alizarin Complexone (AC, 10 mg/mL in 2%NaHCO_3_, pH of 7.4) for 10 min, followed by counterstaining with TO‐PRO‐3. The sections were imaged for these fluorophores in addition to polarized light to visualize the collagen architecture. In the second round, the slides were washed in water following removal of the cover slips, stained with 0.025% toluidine blue (TB), and imaged.

### 2.8 Bone Tunnel Histomorphometry

Images from individual fluorescent channels from the multiple rounds of imaging were aligned in a multi‐image stack using Affinity Photo. The images were then imported into FIJI, and the tunnel region was selected at the interface between the mineralized fibrocartilage (MFC) and bone. Therefore, this region contained the PCL scaffold and the tendon graft (both unmineralized and mineralized regions) but did not contain the adjacent bone. A combination of polarized light and toluidine blue staining was used to delineate the tendon graft from the bone, as previously described [10, 12]. Separate regions of interest (ROIs) were manually defined for the tendon graft (Figure S2B) and the PCL scaffold (Figure S2C) within the bone tunnel. These ROIs were applied to the images containing only AC signal. A histogram was then taken within these ROIs, and a minimum threshold was applied to all samples to quantify the percent positive area of AC signal within these ROIs. All image quantification was performed under blinded conditions with respect to the treatment groups.

### 2.9 Statistics

All statistical analyses were conducted using GraphPad Prism v9. All datasets were tested for normality using the Shapiro–Wilk test (*p* > 0.05) and outliers were removed with the ROUT (*Q* = 1%) test. Fiber diameter and in vivo gene expression were compared via one‐way ANOVA with Tukey's post‐hoc tests (*p* < 0.05). Gene expression results from the in vitro release study were compared via one‐way ANOVA with Tukey's post‐hoc tests to compare control media to SAG concentrations added to directly to the media (3 nM or 300 nM), and two‐way ANOVA with Tukey's post‐hoc tests to compare among the conditioned media treatment groups (*p* < 0.05).

## 3 Results

### 3.1 SAG Incorporation Into the Electrospun PCL Scaffolds Did Not Affect Fiber Alignment or Diameter

**Figure 2:**
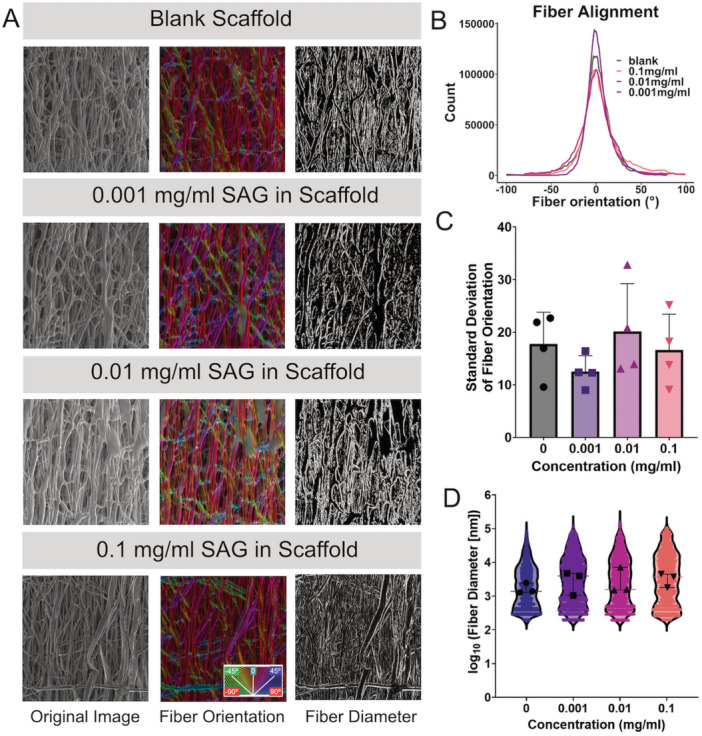
SAG incorporation did not affect fiber orientation or diameter of electrospun PCL scaffolds. (A) SEM micrographs of aligned electrospun scaffolds (B) used to quantify fiber orientation distributions, (C) with corresponding standard deviation of these distributions (*n* = 4), (D) and fiber diameters presented as violin plots (with data points for median values of independent samples in black) of the log‐transformed data from 3 batches of SEM images.

Our initial objective was to determine if the incorporation of SAG affected the intrinsic properties of the scaffold, as alterations to fiber diameter and alignment could affect both the ability of cells to infiltrate the scaffold and their phenotype via biophysical cues from the scaffold architecture [28, 29, 30]. In addition, changes in fiber diameter between scaffolds fabricated with different SAG concentrations would change the surface‐to‐volume ratio and potentially influence the release characteristics of SAG. Therefore, we quantified both fiber alignment and fiber diameter in the fabricated scaffolds. Our analyses revealed no significant differences in either the alignment or diameter of the fibers (Figure 2), indicating that the structure of the scaffolds was consistent between groups and not influenced by the incorporation of SAG.

### 3.2 Delivery of Conditioned Media to bMSCs Increased Gli1 Expression in a Dose‐ and Time‐Dependent Manner

**Figure 3:**
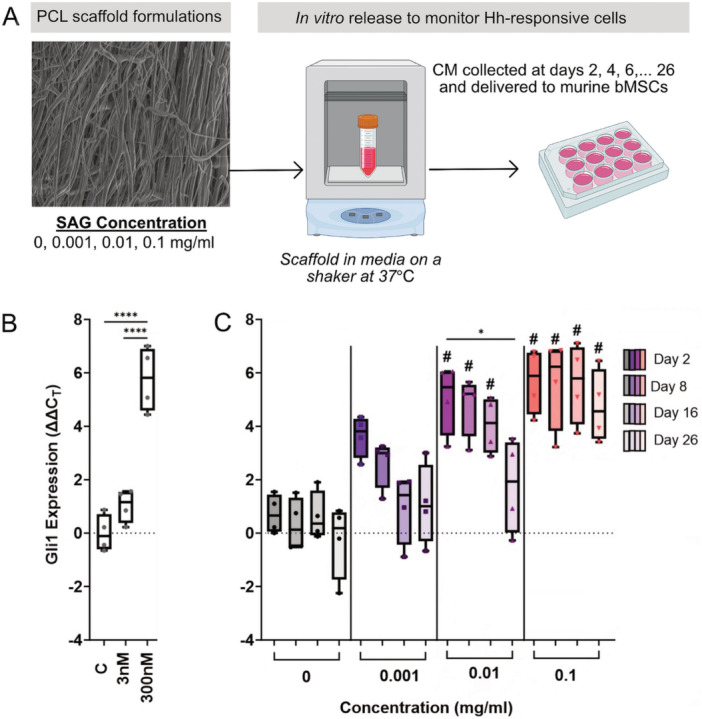
Bioactive SAG was released from the scaffolds in a dose‐ and time‐dependent manner. (A) SAG release study experimental design, where CM released from SAG PCL scaffolds was collected every other day for 26 days. (B) Murine bMSCs were treated with either SAG directly added to the media or (C) CM from the scaffolds collected at different release time points (C) and Gli1 expression was measured via qPCR (*n* = 4 biological replicates/group). *p < 0.05; ****p < 0.0001; #*p* < 0.05 versus 0 mg/mL.

Following the structural analysis of the scaffold, we next determined whether SAG could be released from the PCL fibers while maintaining its bioactivity following the extensive fabrication process and subsequent freezing for scaffold preservation. To do so, CM aliquots collected from SAG‐loaded scaffolds on days 2, 8, 16, and 26 were delivered to the bMSCs for 4 days to assess if SAG released from the scaffolds stimulated Hh signaling (Figure 3A). Results from the aliquots of CM were compared to SAG added directly to the media at multiple concentrations. As anticipated, we found a significant increase in Gli1 expression with 3 nM (~2‐fold) and 300 nM (~60‐fold) SAG added directly to the media (*p* < 0.05, Figure 3B) versus control media. While Gli1 expression did not change in the blank (0 mg/mL) scaffolds compared to baseline, it was significantly higher in cells receiving CM from the 0.01 and 0.1 mg/mL SAG scaffolds compared to 0 mg/mL scaffolds collected on day 2 through 16 (*p* < 0.05, # in Figure 3C). Interestingly, Gli1 expression remained elevated through day 26 (~30‐fold) in the 0.1 mg/mL SAG scaffold group (*p* < 0.05). Of note, the fold change of the 0.1 mg/mL group at early time points was comparable to 300 nM of SAG added directly to the media (Figure 3B). Therefore, SAG released from the PCL scaffolds increased Gli1 expression in bMSCs in a dose‐ and time‐dependent manner.

### 3.3 Localized Delivery of SAG to the Bone Tunnels Increased Hh Signaling Levels

**Figure 4:**
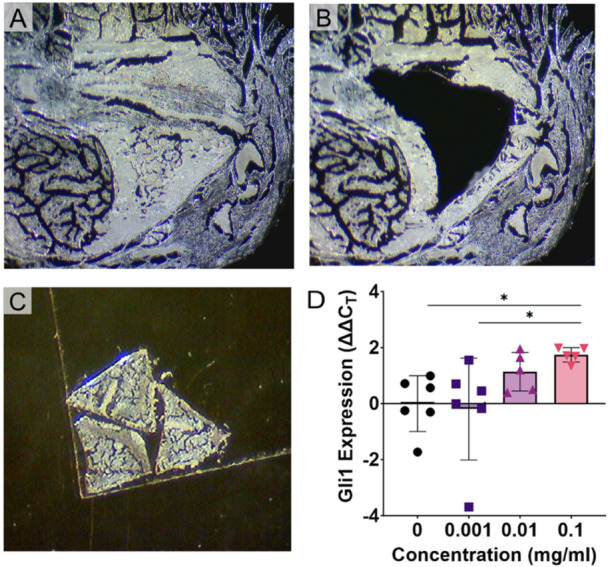
SAG release stimulated Hh signaling in a transverse tibial tunnel (TTT) in vivo model. (A–B) The medullary canal was microdissected out with a 27 G needle from formalin‐fixed cryosections. (C) RNA was isolated from the collected tissue (D) to measure Gli1 gene expression via qPCR (*n* = 5–6/group). *p < 0.05.

Given that in vitro release of SAG from the PCL scaffolds occurred in a dose‐ and time‐dependent manner, we next sought to determine if local release of SAG would stimulate Hh signaling by cells in the bone tunnel and ultimately improve tendon‐to‐bone integration in vivo. Therefore, we combined the scaffolds with the tail tendon graft and then inserted them into the bone tunnel in our TTT surgical model. Similar to the Hh activation in bMSCs following the in vitro release of SAG, we measured expression of Gli1 from cells isolated from within and around the bone tunnels (Figure 4A–C) and demonstrated that SAG release yielded a dose‐dependent effect on Gli1 expression, with the 0.1 mg/mL SAG group resulting in ~3‐fold higher expression, even at 14 days post‐implantation (*p* = 0.009, Figure 4D).

### 3.4 Scaffold‐Mediated SAG Release Increased the Formation of Mineralized Fibrocartilage (MFC) in the Tendon Graft and Mineral Deposition in the Scaffold in the Bone Tunnels

**Figure 5:**
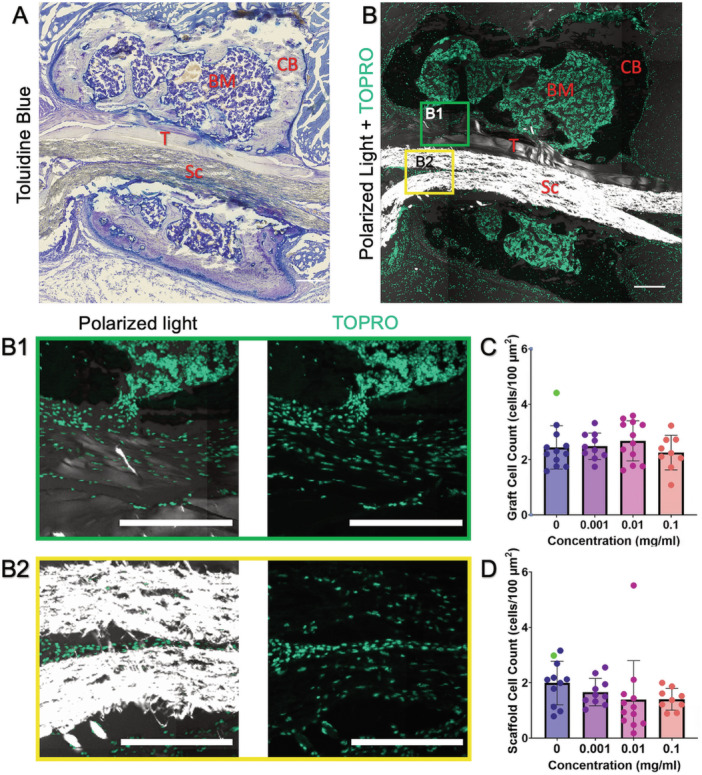
SAG delivery did not affect cell infiltration into the tendon graft or scaffold. (A) Toluidine Blue brightfield image demonstrating the tendon graft (T) and scaffold (Sc) within the bone tunnel. (B) Fluorescent image containing TO‐PRO‐3‐stained nuclei and polarized light imaging highlighting the brighter polarized light signal in the scaffold than the tendon graft. Cells were able to infiltrate the tendon graft (B1, green box) and the PCL scaffold (B2, yellow box). The concentration of SAG did not affect the cell density in the (C) tendon graft or (D) scaffold (*n* = 9–12/group). The sample chosen for A and B was labeled as green dots in the bar plots. Scale = 200 μm. BM, bone marrow; CB, cortical bone.

**Figure 6:**
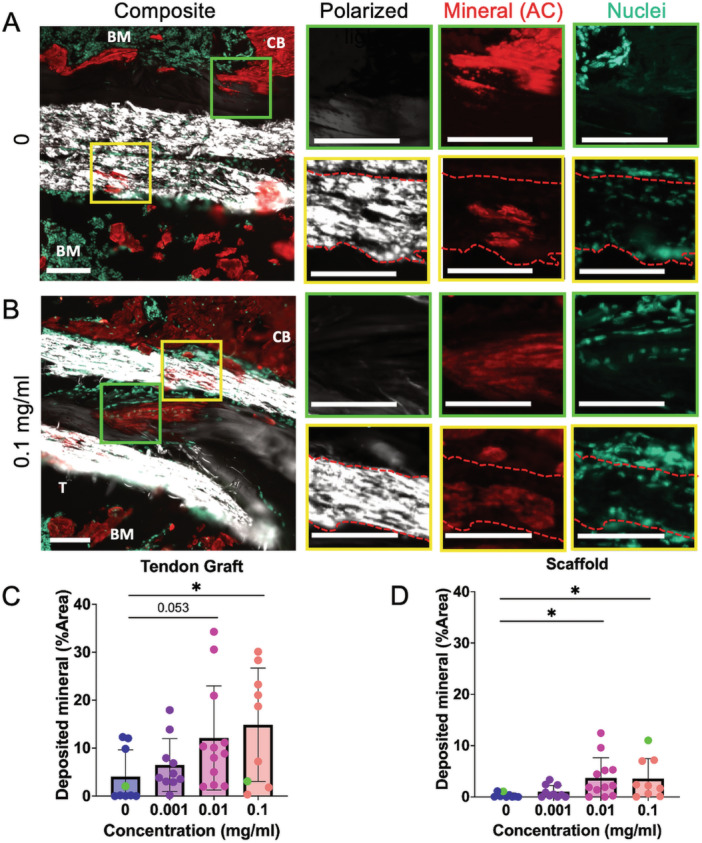
SAG release promoted mineral deposition in the tendon graft and the PCL scaffold. Samples were selected from 0 (A) and 0.1 mg/mL (B) groups to demonstrate mineral deposited in regions of the tendon graft (green boxes, C) and adjacent scaffold (yellow boxes, D) (*n* = 9–12/group). The samples chosen for A and B were labeled as green dots in the bar plots. Scale = 100 μm. BM, bone marrow; CB, cortical bone; T, tendon. *p < 0.05.

We next determined if the increased expression of the downstream target Gli1 resulted in improved tendon‐to‐bone integration, similar to our previous findings using systemic administration [12]. Therefore, we measured the cell infiltration (Figure 5) and deposited mineral area (Figure 6) in the tendon graft and the PCL scaffold from adjacent transverse sections. Since this was the first time that our group implanted a PCL scaffold into the bone tunnel, we first assessed whether the PCL scaffold was receptive to cellular invasion or if it would impair the ability of cells to infiltrate the bone tunnel. We found that cells were able to infiltrate the PCL graft (Figure 5D) and importantly were able to deposit matrix and mineral (Figure 6). Since Hh signaling promotes proliferation in a number of cell types [12, 31, 32, 33, 34], it is conceivable that localized SAG delivery could promote increased cell infiltration into the tendon graft and scaffold by stimulating proliferation of the bMSC progenitor pool that gives rise to the tunnel integration process [10]. Therefore, we quantified cell density within both the tendon graft and PCL scaffold and did not find a significant effect of SAG delivery on cell infiltration (Figure 5C–D). We next measured mineral deposition within both the tendon graft, indicative of MFC production, and in the adjacent PCL scaffold. The mineralized fibrocartilage within the tunnels differs from the surrounding bone in that it exhibits a proteoglycan‐rich staining in the surrounding matrix (Figure S3). We found that SAG delivery promoted mineral deposition in a dose‐dependent manner (Figure 6C–D). Specifically, the 0.1 mg/mL group had significantly higher MFC formation in the tendon graft (*p* = 0.044, Figure 6C), while both the 0.01 mg/mL (*p* = 0.048) and 0.1 mg/mL (*p* = 0.047) groups had higher mineral deposition in the scaffold (Figure 6D). In addition, we did not observe any adverse effects in the mice, such as hair loss or ectopic mineralization, as seen previously by our group with systemic agonist injections and genetic over‐expression models [12]. These findings indicate that SAG released locally from the PCL scaffold stimulated the resident cells to produce higher levels of MFC in the tendon‐to‐bone attachments during the tunnel integration process without off target effects.

## 4 Discussion

Tendon‐to‐bone integration is a complex and coordinated process that culminates in the formation of zonal fibrocartilaginous attachments, essential for functional graft incorporation [10]. Previous studies from our group and others have demonstrated that the Hh pathway is not only active during tendon‐to‐bone repair but can also positively regulate the healing process. In the current study, we sought to leverage the Hh pathway to improve the tendon‐to‐bone integration process by developing a novel approach using SAG‐loaded electrospun PCL scaffolds to locally stimulate the pathway in cells in the bone tunnels. Our data indicate that the electrospun PCL scaffolds maintained consistent fiber alignment and diameter irrespective of SAG incorporation. This stability in scaffold architecture is crucial as it implies that the biophysical cues (fiber size, spacing, and alignment) of the scaffold architecture are not influenced by the incorporation of SAG. The in vitro release of SAG from the scaffolds resulted in a dose‐ and time‐dependent increase in Gli1 expression in bMSCs in culture, underlining the potential for SAG delivery to sustain Hh activity over extended periods. Notably, the 0.1 mg/mL SAG concentration achieved the most prolonged elevation of Gli1 expression, suggesting it as an optimal dose for sustained signaling. In Vivo studies further validated these findings, revealing that localized SAG delivery significantly increased Gli1 activity and promoted mineralized fibrocartilage formation with no evidence of ectopic mineralization, which previously occurred in our genetic over‐expression mouse model [12]. The observed dose‐dependent effects underscore the importance of precise SAG dosing to achieve desired biological outcomes while avoiding off‐target effects seen with systemic delivery. Enhanced mineral deposition in both the tendon graft and the PCL scaffold indicates improved integration and mineralization, critical for graft functionality and longevity.

The results of this study leverage the therapeutic potential of the Hh signaling pathway by translating mechanistic findings from prior research into a novel therapy. Several studies [7, 8, 11, 12, 35] previously demonstrated the importance of the Hh pathway in tendon‐bone healing. For instance, a recent study [8] illustrated that Hh signaling directed cell differentiation and rotator cuff healing as genetic activation of the pathway after injury promoted both type X collagen and Osterix production in the injury site. Additionally, Luzzi et al. [7] demonstrated that locally activating the Hh pathway with delivery of a Hh agonist from implanted microspheres enhanced rotator cuff tendon‐to‐bone healing. Similarly, we demonstrated that both genetic activation and systemic administration of a Hh agonist increased the formation of mineralized fibrocartilage‐containing zonal tendon‐to‐bone attachments in an ACL reconstruction model. In the current study, our work improves upon the previous findings from our group using systemic agonist delivery by developing a novel scaffold‐mediated delivery system to provide local delivery of SAG via electrospun PCL scaffolds. This approach improves mineral deposition both in the tendon graft and the implanted PCL scaffold, thereby promoting better tendon‐to‐bone integration in a dose‐dependent manner. Importantly, we did not observe the adverse effects that were observed previously with systemic delivery of the agonist or genetic activation in specific cell types [12]. Therefore, our approach of using a scaffold to locally release SAG provides a more controlled and sustained activation of the signaling pathway while minimizing the off‐target effects from systemic delivery.

The use of drug delivery systems to enhance tendon‐to‐bone healing has gained considerable attention, particularly through the localized delivery of signaling molecules like growth factors or small‐molecule drugs. While growth factors like Indian Hedgehog and Sonic Hedgehog are potent inducers of tissue regeneration, their clinical application is often limited by challenges such as short biological lifespan under physiological conditions, difficulty loading into delivery systems, and higher cost [36, 37]. In contrast, small molecules like SAG offer a more practical alternative due to their increased stability, ease of incorporation into delivery systems, and potential for even more sustained release. Similar to the current study, previous work has demonstrated that small‐molecule drugs can be effectively delivered using electrospun scaffolds [21, 38, 39], providing a controlled and sustained release of therapeutic agents directly at the site of injury, improving repair outcomes [40, 41]. This approach not only simplifies the delivery process but also enhances the precision of treatment, reducing side effects from systemic delivery while also providing therapeutic concentrations at the site of injury.

This study is not without limitations. While the transverse tibial tunnel model allowed us to test our hypothesis, its clinical relevance is limited. It was chosen for its higher throughput and ability to make bilateral comparisons, while our ACLR model is more clinically relevant and will be the focus of future work using this optimized system. Additionally, while the in vitro studies provided insights into the release profile of SAG from the PCL scaffolds, understanding the exact distribution and concentration within the scaffold was not evaluated. Slight variations in SAG incorporation spatially within the scaffold could alter regional concentration in the bone tunnels in vivo. Our group is developing novel scaffolds to address regional variations that could occur to further localize the SAG to specific regions of the scaffold. Additionally, although Gli1 is a well‐established and reliable readout of Hedgehog pathway activation, we acknowledge that evaluating additional downstream components would provide a more comprehensive assessment of pathway activity. Future studies will aim to include such markers.

## 5 Conclusion

Results from this study demonstrate the efficacy of locally delivering SAG to stimulate the hedgehog pathway in both in vitro and in vivo contexts. This approach enhances tendon‐to‐bone integration following surgery by maintaining the activation of the hedgehog pathway to promote fibrocartilage production and mineral deposition. The dose‐ and time‐dependent enhancements observed with SAG‐loaded scaffolds underscore the therapeutic potential of such systems in promoting robust tendon‐to‐bone healing. These outcomes are pivotal for enhancing the structural integrity and functionality of the tendon‐to‐bone repair site, ultimately contributing to better surgical outcomes. Our research sets the stage for future work aimed at refining SAG delivery and exploring additional factors that could augment scaffold efficacy. Transitioning these findings into more clinically relevant tendon‐to‐bone integration models in larger species that more closely replicate the size and forces experienced by humans is essential for clinical translation. Ultimately, our findings will pave the way for advanced strategies in tendon‐to‐bone integration therapy, leveraging the hedgehog pathway to enhance tissue repair and regeneration.

## Author Contributions

Experiment conception and design completed by Jonathan Marcelin, Rashad Madi, Timur B. Kamalitdinov, Xi Jiang, Robert L. Mauck, Andrew F. Kuntz, Nathaniel A. Dyment. Experiment execution completed by Jonathan Marcelin, Rashad Madi, Dong Hwa Kim. Data analysis and interpretation completed by Jonathan Marcelin, Rashad Madi, Timur B. Kamalitdinov, Xi Jiang, Andrew F. Kuntz, Nathaniel A. Dyment. The paper was written and edited by Jonathan Marcelin, Rashad Madi, Timur B. Kamalitdinov, Xi Jiang, Dong Hwa Kim, Robert L. Mauck, Andrew F. Kuntz, Nathaniel A. Dyment. All authors approved the submitted manuscript.

## Supporting information


**Figure S1:** Hh agonist (SAG) released from PCL scaffold increased downstream Gli1 expression. **Figure S2:** Images were imported into FIJI (ImageJ), and separate regions of interest (ROIs) were manually defined for the tendon graft area (B) and PCL scaffold area (C) within the bone tunnel. **Figure S3:** The mineralized fibrocartilage within the tunnels differs from the surrounding bone by exhibiting a proteoglycan‐rich staining in the pericellular matrix (A).
