# Supplementary material for: Localized Delivery of a Small Molecule Hedgehog Agonist via Poly(ε‐caprolactone) Scaffolds Enhances Tendon‐to‐Bone Integration
Source: J Orthop Res. 2026 Mar 30;44(4):e70183. doi: 10.1002/jor.70183 (PMC13036285; doi:10.1002/jor.70183)
Supplement: Supplementary file 1 — Figure S1: Hh agonist (SAG) released from PCL scaffold increased downstream Gli1 expression. Figure S2: Images were imported into FIJI (ImageJ), and separate regions of interest (ROIs) were manually defined for the tendon graft area (B) and PCL scaffold area (C) within the bone tunnel. Figure S3: The mineralized fibrocartilage within the tunnels differs from the surrounding bone by exhibiting a proteoglycan‐rich staining in the pericellular matrix (A). [file JOR-44-0-s001.pdf]

Supplemental Materials

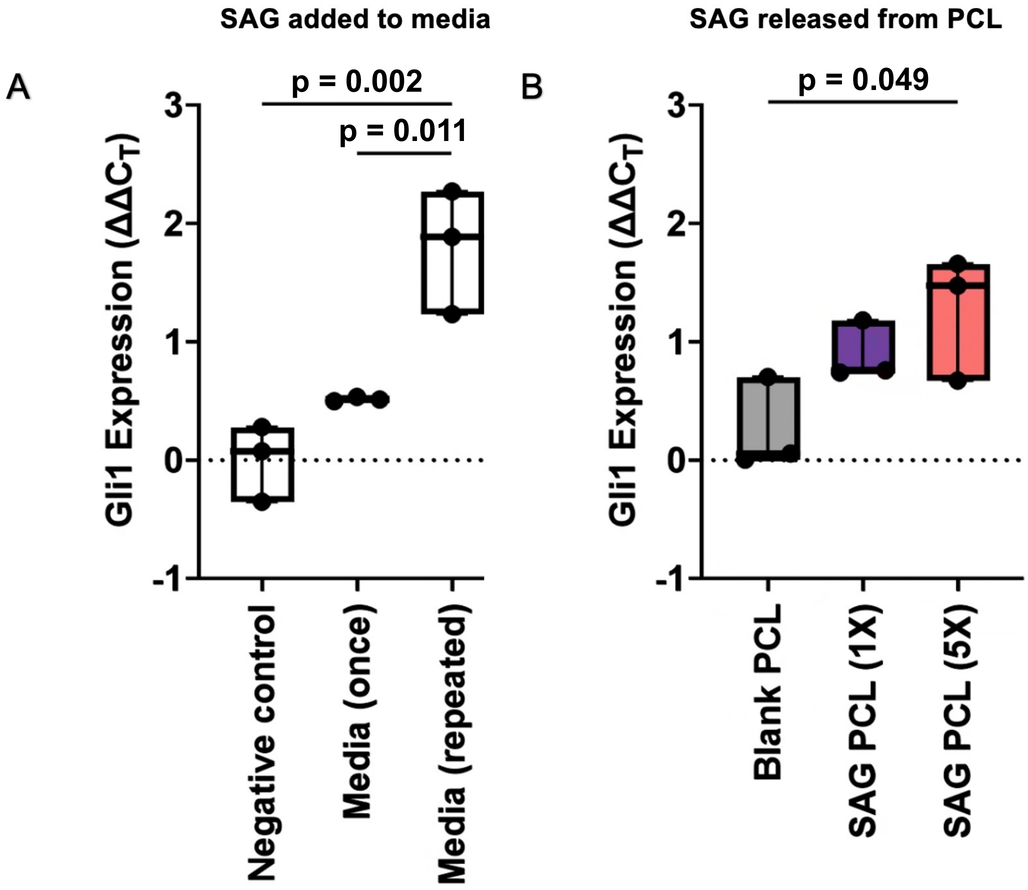

Fig. S1. Hh agonist (SAG) released from PCL scaffold increased downstream Gli1 expression. We conducted a preliminary study to confirm that SAG loaded into PCL nanofibers remains bioactive upon release. As a baseline, we tested the effect of SAG (3nM) added directly to the media of murine bMSC cultures either once on day 0 or repeatedly on days 0, 2, and 4 via media changes. We measured expression of Gli1 on day 6 and found a nearly 4-fold increase in Gli1 expression compared to the negative control (A; p=0.002 for negative control vs media-repeated, and p=0.011 for media-once vs media-repeated, n = 3 biological replicates/group). Next, we loaded SAG into the PCL spinning solution at 0.1mg/ml. We cut 10x10 mm patches of scaffold and placed them in cell culture inserts while bMSCs were cultured in the well below for 6 days. We tested 3 scaffold conditions: 1 layer of blank (0mg/ml), 1 layer (1X) of 0.1mg/ml, and 5 layers (5X) of 0.1mg/ml (B). We found a significant increase (p=0.049) in Gli1 expression in the 5X SAG PCL group compared to the blank PCL (n = 3 biological replicates/group). Gene expression results were compared via one-way ANOVA with Tukey's post-hoc tests.

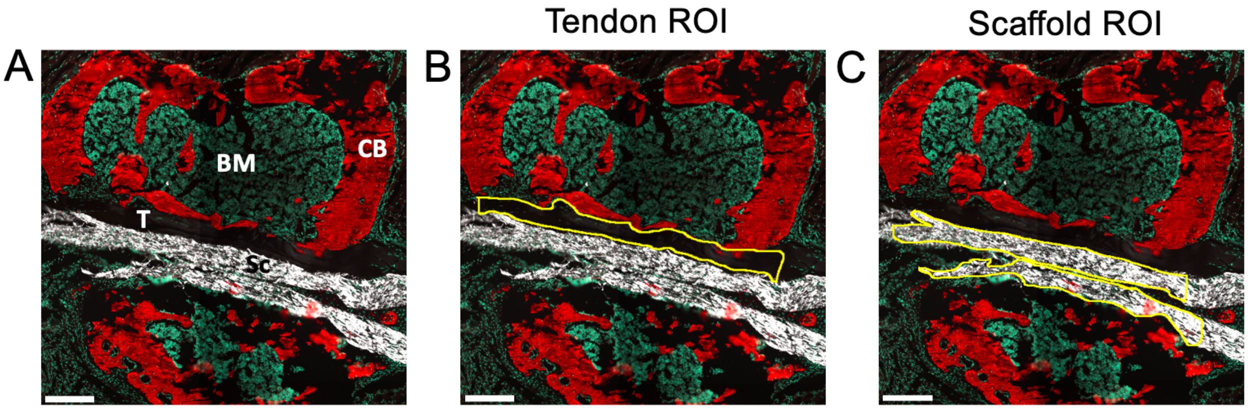

Fig. S2. Images were imported into FIJI (ImageJ), and separate regions of interest (ROIs) were manually defined for the tendon graft area (B) and PCL scaffold area (C) within the bone tunnel. The boundaries between these regions and the surrounding bone were identified using a combination of polarized light imaging and toluidine blue staining. Histograms were then generated for each ROI to independently quantify the percent area positive for AC signal. The sample used in this figure fell in the 0 mg/ml group (mean ± SD, 4.06 ± 5.58%), with 2.04% MFC. For comparison, the amount of MFC in the other groups were 6.47 ± 5.50%, 12.12 ± 10.86%, and 14.87 ± 11.82% for the 0.001, 0.01, and 0.1 mg/ml groups respectively. Scale = 200µm. BM: bone marrow; CB: cortical bone; T: tendon; Sc: scaffold.

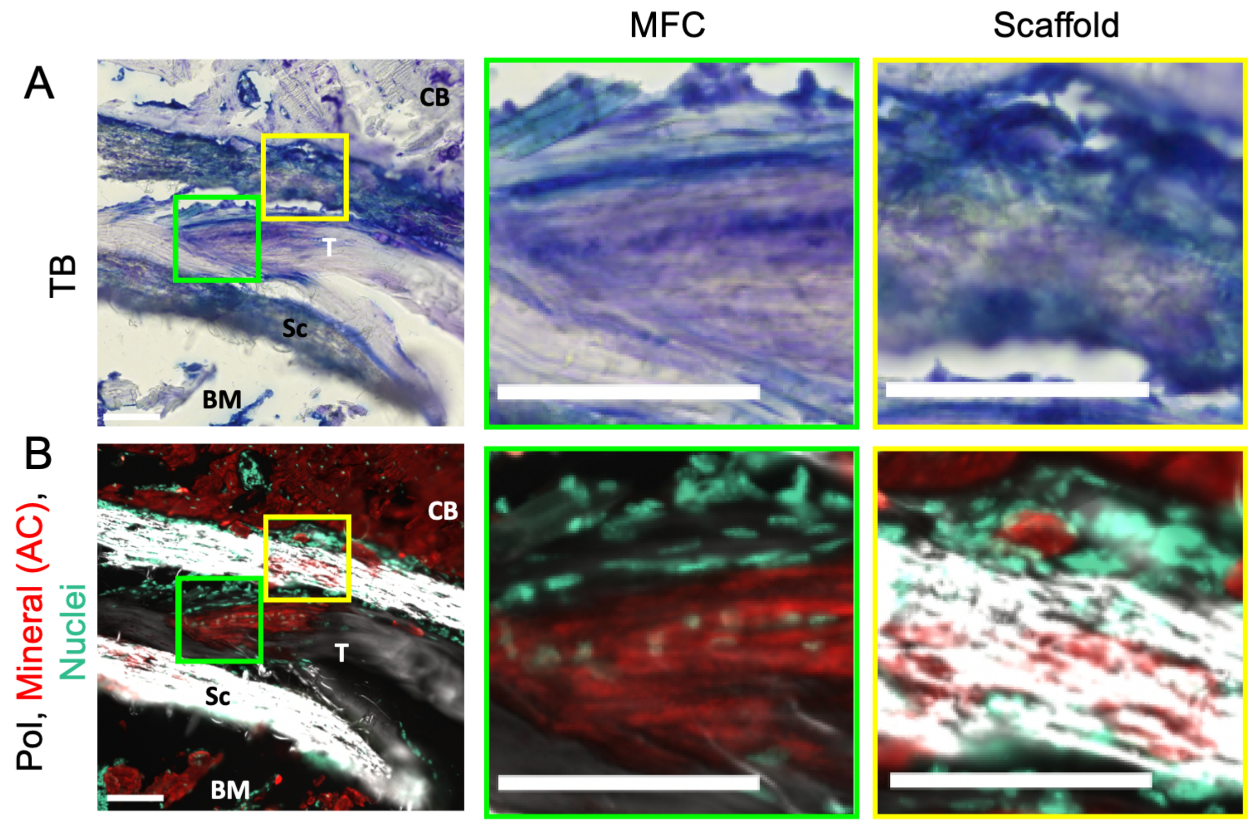

Fig. S3. The mineralized fibrocartilage within the tunnels differs from the surrounding bone by exhibiting a proteoglycan-rich staining in the pericellular matrix (A). These proteoglycan-rich areas align with the Alizarin complexone (AC) staining in the tendon (MFC; green boxes, B) and in the scaffold (mineral deposition; yellow boxes, B). Scale = 100µm. BM: bone marrow; CB: cortical bone; T: tendon; Sc: scaffold.
